# Use of processed nerve allograft to relieve pain and restore function in individuals with painful upper limb neuroma—a case series of five individuals

**DOI:** 10.3389/fsurg.2026.1819234

**Published:** 2026-07-30

**Authors:** Lars B. Dahlin, Erika Nyman

**Affiliations:** 1 Department of Translational Medicine – Hand Surgery, Lund University, Clinical Research Center (CRC), Malmö, Sweden; 2 Department of Hand Surgery, Skåne University Hospital, Malmö, Sweden; 3 Department of Biomedical and Clinical Sciences, Linköping University, Linköping, Sweden; 4 Clinical Department of Hand Surgery, Plastic Surgery and Burns, Region Östergötland, Linköping University Hospital, Linköping, Sweden; 5 Department of Orthopedic and Hand Surgery, Faculty of Medicine and Health, Örebro University, Örebro, Sweden

**Keywords:** iatrogenic nerve injury, nerve injury, nerve reconstruction, neuroma, pain, processed nerve allograft

## Abstract

**Introduction:**

A traumatic nerve injury that is improperly treated or left untreated may result in a painful neuroma with compromised sensory and motor function. Affected individuals might be hesitant to donate a healthy nerve for an autologous nerve graft procedure. The use of processed nerve allografts (PNA) to reconstruct nerve injuries with painful neuromas is less often discussed. We aim to offer perspectives on treating painful neuromas with PNA when autograft reconstruction is considered not an option.

**Methods:**

This series involves five individuals with neuroma formation and varying pain and functional impairment after injuries to different upper limb nerves, i.e., two median nerves at the elbow level, two digital nerves (thumb and little finger), and an isolated ulnar motor branch. Surgery was indicated for pain, not essentially for functional improvement. Treatment decisions were based on patients' symptoms and disability, injury location and nature, time since injury, and previous surgeries. No other options were considered other than neuroma reconstruction with PNA (length of PNA 25-70 mm). Various clinical and electrophysiological methods were used to evaluate outcomes.

**Results:**

Four out of five individuals experienced improvement after surgery in pain at rest and during activity, but they still had pain during percussion (Tinel's test). Some sensory and motor functions improved, as assessed by clinical and electrophysiological observations. One individual reported ongoing problems, mainly pain and sleep difficulties, and was subsequently referred to a pain clinic.

**Discussion:**

Using PNA for reconstruction may be a suitable solution to relieve pain and improve some function in individuals with painful upper limb neuromas, when autologous nerve grafting is not an option, and even when conducted a long time after the initial nerve injury.

## 1 Introduction

Peripheral nerve injuries are preferentially repaired or reconstructed immediately, or as soon as possible after the injury, since timing of surgery is crucial, particularly for major nerve trunks, but is less important for digital nerves (1–4). Residual problems after nerve surgery, regardless of the treatment method, are often due to the formation of various types of neuromas. Such problems may include loss of motor and sensory function, cold sensitivity, and pain (5–9). Neuropathic pain after neuroma formation, with its complex pathophysiology (10, 11), is difficult to treat irrespective of pharmacological treatments (12) or despite the surgical method used (5, 13–15). Use, and even addiction to opioids and other pharmacological substances may occur, but have been reported to be reduced by surgery, in nerve injuries and neuromas (16–18).

The options for treating symptomatic neuromas vary depending on the affected nerve and the extent of the injury. Several surgical procedures are available, but none appear optimal (13). In certain neuroma cases, when conventional methods, such as nerve repair or reconstruction, are not suitable or are declined by the affected individual, a processed nerve allograft (PNA) may be an alternative (19, 20), as in other nerve injuries (21, 22). However, the use of PNA in neuroma treatment in the upper limb is less well documented (5, 23, 24), where pain related to the neuroma formation may impact the quality of life because of the motor and sensory dysfunction, risk for involved psychological factors and sleeping problems, as well as the influence of socioeconomic position (7, 13, 23). The outcomes of surgery using PNAs in foot and ankle neuroma have been presented. A neuroma in the lower limb may have broader impacts on an individual's functioning and introduce additional aspects of daily life (25, 26). One aspect to consider before performing neuroma surgery is the time between the initial injury and neuroma surgery, the number of previous surgeries, the affected nerve, and the cause of the injury (e.g., iatrogenic nerve injury) (24). Our aim was to present five individuals of different neuromas, causing pain and discomfort, treated with PNAs long time after the initial injury, and in two individuals, with previous nerve surgery. In the five individuals, no other treatment options than PNAs were considered applicable.

## 2 Case descriptions

The present series comprises five individuals with varying degrees of pain, discomfort and disability following injuries to different upper-limb nerves, accompanied by neuroma formation. The indication for surgery was, in all individuals, pain-discomfort, and not primarily an intention to improve function. Based on the problems described by the individuals, the location and nature of the nerve injury, the time after the initial injury, and in two individuals, previous attempts to treat the injury, no other surgical or medical treatment options were considered as applicable than reconstruction of the neuroma using a PNA. Accordingly, different clinical and electrophysiological methods were used to evaluate the outcomes based on the location and extent of the specific nerve injury. Below is a summary of the five surgically treated individuals presented in the text. Detailed descriptions of demography and treated nerve, details of timing and nerve reconstruction, as well as subjective and objective findings as outcomes of each individual, are presented in Table 1.

**Table 1:** Summary of five individuals with different neuromas after nerve injuries in the upper limb reconstructed with processed nerve allografts (PNA) and follow-up.

Sex and age at surgery	Cause(s) of injury	Earlier surgery	Subjective surgical outcome (incl. pain and Tinel's test)	Objective sensory function postoperatively	Objective motor function postoperatively
Follow-up time	Indication for surgery and preoperative symptoms and findings	PNA surgery and concomitant procedure(s)			
Injured nerve					
Duration until PNA surgery *(from injury or previous surgery)*					
Male, 20–30 yrs	Explosion injury with concomitant injuries (radial nerve injury; elbow)	No previous surgery	Overall improved function	Index finger pulp: 2-PD 8 mm	Return of flexion in thumb, index, and long finger (M4-), and some FCR function (M2)
72 mo			No pain at rest (0/100) or activity (0/100), still pain at Tinel's test (100/100) at proximal suture line		
Median nerve at elbow	Severe pain in medial upper arm, complete loss of median nerve function (EMG confirmed), palpable neuroma, pain at Tinel's test (100/100)	PNA (length 70 mm; thickness 5 mm) to distal nerve end	Return of some sensation and motor function, remaining radial nerve injury dysfunction		Neurography and EMG (36 mo): 50% of motor amplitude, no sensory amplitude, PT = signs of reinnervation, APB = sparse reinnervation
60 mo from injury					
Female, 40–50 yrs	Sharp cut injury	Two previous neuroma surgeries	Improved function	Thumb pulp: SWM 4.31	NA
26 mo			No pain at rest (0/100), light activity (0/100), or pain at Tinel's test (0/100)		
Radio-volar digital nerve in thumb	Deep pressure pain during activity (40–50/100)	PNA (length 50 mm; thickness 2–3 mm) to distal nerve end	Return to ordinary work full-time, some persisting radiating pain at heavy load/pressure at work		
Previous surgery 84 and 24 mo earlier			No use of analgesics		
Male, 20–30 yrs	Iatrogenic injury during surgery for ganglion distal to hook of hamate	Two previous surgeries (ganglion)	Improved grip and manual dexterity	All finger pulps: SMW 2.82	IOD1 M4- AP M3
39 mo					
Deep branch of ulnar nerve	Impaired grip function, fatigue, clawing ring-little fingers, pain-discomfort, intrinsic denervation/atrophy (EMG confirmed)	PNA (length 35 mm; thickness 2 mm) to distal nerve end.	Normal sensation, less fatigue, less pain-discomfort, better pulp-to-pulp pinch, remaining atrophy in ulnar part of hand (clawing)		EMG (24 mo): no signs of reinnervation
Initial surgeries 39 and 21 mo earlier		AIN transfer to motor branch in forearm			
Male, 50–60 yrs	Iatrogenic injury during surgery for elbow problems	Previous nerve reconstruction with sural nerve 4 months post-injury	Subjectively unchanged.	Unchanged	Unchanged
13 mo		PNA (length 70 mm; thickness 5 mm) to distal nerve end			
Median nerve at elbow	Severe burning pain at rest (100/100), pain at Tinel's test (100/100), severe cold sensitivity, sleep problems, sensory loss median nerve, some remaining opposition and pronation, preserved FDP III–V	Tendon transfer FDP II=>III Intact fascicles to PT	Remaining neuropathic pain at elbow and in median nerve innervated area, pain at Tinel's test (75/100), allodynia (70–80/100), sleeping problems, still on sick leave, declined further surgery		
12 mo after last surgery	Neurography/EMG: some APB function, no PT function, no sensory response	No AIN reconstruction. Later decision about FPL transfer	Referral to pain clinic		
Male, 60–70 yrs	Saw injury	No earlier neuroma surgery (only acute nerve suture)	Overall improved function.	Little finger pulp: SMW 3.61 2-PD 12 mm	NA
41 mo					
Ulnar-volar digital nerve in little finger	No pain at rest (0/100), pain at activity (75/100), pain at Tinel's test (95/100), allodynia (70/100), impaired sensation (2-PD not measurable), flexion contracture PIP joint	PNA (length 25 mm; thickness 2 mm) to distal nerve end	No pain at rest (0/100), pain at activity (30/100), pain at Tinel's test (50/100), no allodynia, improved sensation, remaining flexion contracture		
14 mo from injury	Suboptimal effect of analgesics		No use of analgesics		

PNA, processed nerve allograft; Mo, months; Yrs, years; 2-PD, two-point discrimination (pulp); SWM, Semmes-Weinstein monofilament (pulp); NA, not applicable; FCR, flexor carpi radialis muscle; PT, pronator teres muscle; APB, abductor pollicis brevis muscle; ROM, range of motion; EMG, electromyography; IOD 1, first dorsal interosseous muscle; AP, adductor pollicis muscle; FDP, flexor digitorum profundus muscle; FPL, flexor pollicis longus muscle; AIN, anterior interosseous nerve; PIP, proximal interphalangeal joint.

### 2.1 Demography

Five individuals with neuroma after a nerve injury in the upper limb (four men and one women; age range 20–70 years; only age intervals presented for anonymity) were included from two departments in Sweden (Clinical Department of Hand Surgery, Plastic Surgery and Burns, Linköping University Hospital, Sweden and Department of Hand Surgery, Skåne University Hospital, Sweden) and reconstructed with a PNA without any connector-assisted repair of the attached nerve ends nor a protector wrapped around the allograft or repair sites [such as Axoguard® Nerve Protector (24)]. The PNAs (Avance® Nerve graft; Axogen Inc, Fl, US) were bought by the hospitals in Malmö and Linköping, Sweden, through the Danish supplier (Innosurge A/S, Randers, Denmark). The producer of the PNAs or the Danish supply company had no impact on the indication of surgery, evaluation of the treated individuals, or the content of the present manuscript. All individuals approved the inclusion of their data in the study.

### 2.2 Reconstructed nerves, time since injury, and previous surgical procedures

The individuals involved different nerve injuries with two major nerve trunks (median nerve), two digital nerves (little finger and thumb), and one deep branch of the ulnar nerve (Table 1). Three out of the five individuals had a traumatic nerve injury, and two individuals had an iatrogenic nerve injury as a cause of neuroma formation with pain. The individual with the iatrogenic injury to the deep branch of the ulnar nerve had pain-discomfort due to motor dysfunction. The time interval between the first injury and the PNA reconstruction ranged from 14 to 84 months, with two individuals having one or two prior nerve reconstruction procedures, respectively.

### 2.3 Reconstruction with PNA

**Figure 1:**
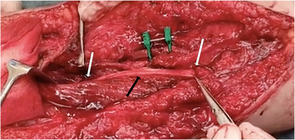
Image of a processed nerve allograft (PNA; black arrow) used to reconstruct a median nerve at the elbow (individual 1; elbow to the right) with the two repair sites marked with white arrows. Permission to reuse the image from a video was granted by Elsevier Inc., Netherlands.

**Figure 2:**
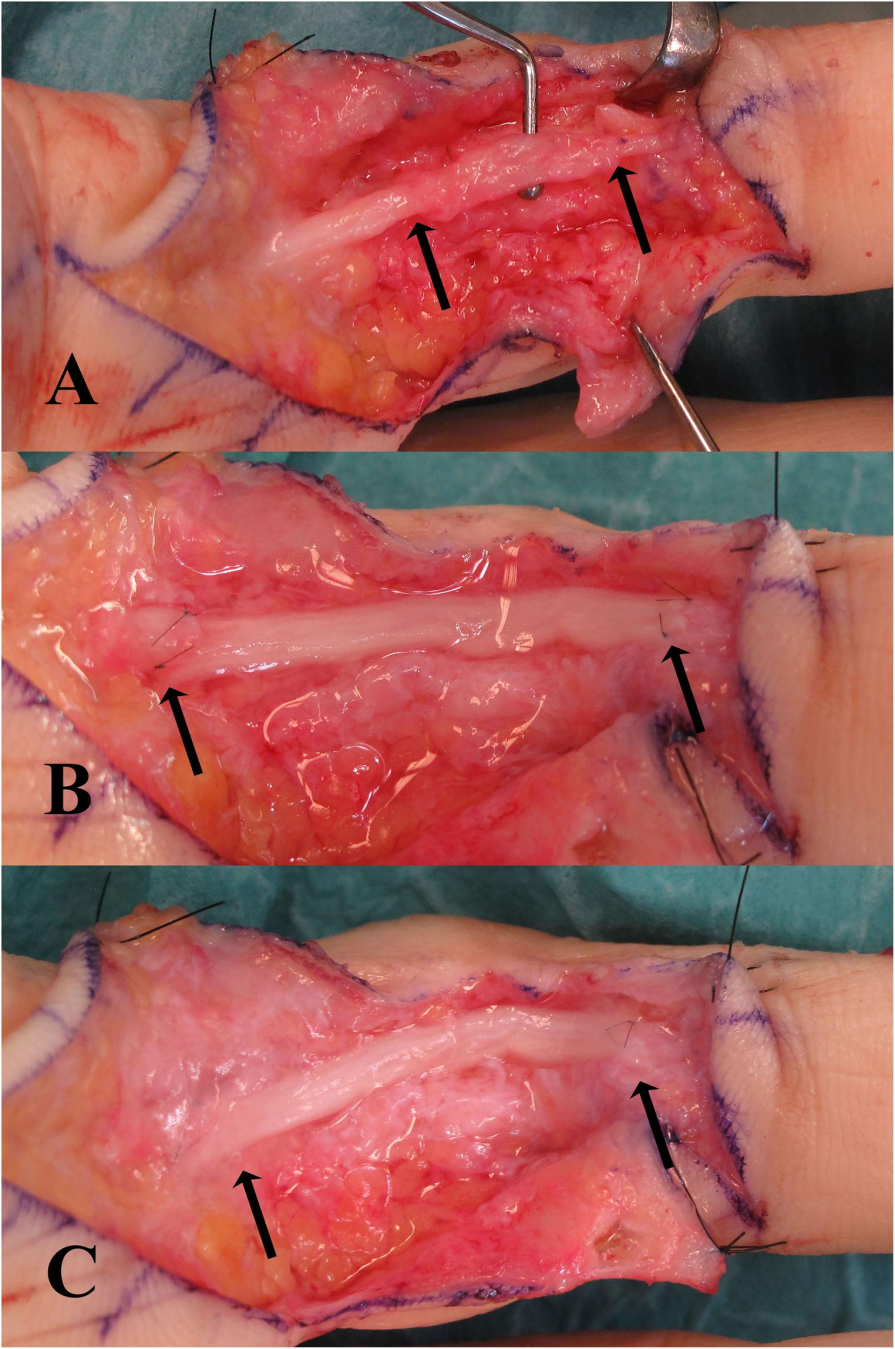
Image of a digital nerve reconstructed with a processed nerve allograft (PNA; individual 5). The long neuroma-in-continuity **(A)** lifted and delineated with two black arrows (finger pulp to the right). The PNA in place **(B,C)** with the proximal and distal suture lines (two at each repair site) marked with arrows **(B)** and then covered with Tisseel® [marked with black arrows in **(C)**].

The lengths of the PNAs ranged from 25 to 70 mm, and three individuals had a thin PNA (2–3 mm), and two had a thick (5 mm) PNA. The follow-up ranged from 13 to 72 months. By the use of a microscope and micro-instruments, all PNAs were applied and secured with a single 8-0 or 9-0 suture at each end to the proximal and distal nerve ends, and then fibrin glue (Tisseel®, Baxter, Kista, Sweden) was applied around each site of repair as in nerve reconstruction with autologous nerve grafts (Figures 1, 2).

### 2.4 Outcome of surgery

Four out of five individuals reported an improved pain-discomfort situation, and one individual described unchanged symptoms with persistent pain, sleeping problems, and decline of further surgery; thus, he was referred to a pain clinic. Different outcomes by surgery, depending on the preoperative symptoms and affected nerve, were narrated with better grip function (deep branch of ulnar nerve; Table 1) and less pain (digital and major nerve injuries), although some residual pain, such as pain at load, and percussion of the reconstructed area with Tinel's sign remained (Table 1). Some sensation was restored in these individuals in which a distal nerve end was present at surgery and could be attached to the PNA. In the individual with a reconstruction of the deep branch of the ulnar nerve, having more pain-discomfort because of the affected motor nerve branch, some clinical function was observed in the first dorsal interosseous (IOD1) and in the adductor pollicis (AP) muscles at 39 months, with improved grip and manual dexterity, although no reinnervation of those muscles was detected by Electromyography (EMG) at 24 months (Table 1).

### 2.5 Ethics

The study was planned and conducted in accordance with the ethical principles of the Declaration of Helsinki (27) and was approved by the Swedish Ethical Review Authority (No 2023-01957-01). All individuals provided informed consent.

## 3 Discussion

In the present retrospective follow-up, five individuals were surgically treated with nerve reconstruction using PNAs attached to a distal nerve end with the intention of particularly reducing pain and discomfort from a locally painful neuroma. Four out of five individuals improved after the surgery regarding pain at rest and at activity but still had pain at percussion (Tinel's test), while one individual reported persistent problems and was later referred to a pain clinic.

The surgical procedures were performed for different reasons, a rather long time after the initial nerve injury, and in three of five individuals, other surgical procedures had been performed earlier without improvement. In no individual was nerve reconstruction with autologous nerve grafts considered. The reasons were the time since injury, the low potential for functional recovery, the risk of donor-site morbidity, prior nerve reconstructions with autologous grafts, or the individual's reluctance to undergo such a procedure. Thus, the indication for using a PNA was applicable despite the discussion of their utility and efficacy regarding the length of the reconstructed nerve defect and the diameter of the PNA (26, 28–30). It was worthwhile, as reported by four of five individuals, to reconstruct the nerve injury, even if previous surgical neuroma surgeries had been performed. Thus, in agreement with a previous report on chronic pain using PNA to alleviate such a problem in mainly sensory and some mixed sensorimotor nerves, with various time intervals between injury and reconstruction and variability in PNA length (24, 26, 31), the pain-discomfort improved in the present four individuals. PNAs can also be used if a distal nerve end is not present. using the concept of processed nerve “allograft to nowhere” (31–33). This concept has been experimentally developed in PNAs and in other acellular models (19, 20, 34, 35), where reduced infiltration and accumulation of T-cells and macrophages may be suggested mechanism(s) for limited nerve regeneration in long acellular nerve allografts and in other types of nerve graft models (36, 37).

The intention of surgery was preoperatively only to reduce or abolish the pain by allowing the axons from the proximal nerve end to regenerate into a long PNA; i.e., interpreted as a “blind” PNA even if the PNA was connected to a distal nerve end. Axonal outgrowth in a “blind” PNA without a distal nerve end is an option for treating neuroma based on experimental studies (19, 20, 23, 35), which may also involve a xenograft approach (38, 39). The pathophysiology underlying this surgical option is that the axonal outgrowth is limited in a controlled manner and that spontaneous activity is reduced in the sensory neurons (34, 40). Optimal axonal outgrowth is achieved when a distal nerve segment is attached, as was possible in the present individuals although in some of the individuals the nerve defect was extensive. In such a situation, Schwann cells can migrate into any acellular tissue or conduit and approach the growing axons that travel alongside them from the proximal nerve end (41). An optimal nerve regeneration requires that angiogenesis with the formation of new blood vessels in the PNAs is possible, a process that is impaired in long acellular nerve allografts with an associated lower infiltration and accumulation of T-cells (36). Macrophages, crucial cells for nerve regeneration, may also be of relevance in acellular nerve grafts and in PNAs (37, 41). Still, growth-promoting substances like laminin, are present in acellular nerve allografts (39).

Recently, tacrolimus (FK506) was reported as an adjunct to modulate the regenerative microenvironment in rat acellular nerve allografts, resulting in improved nerve regeneration (42), in contrast to its effects of FK506 in autologous nerve grafts in rats (43). In one of the present individuals with median nerve reconstruction, the PNA was attached to a distal nerve end, and the axons could bridge the PNA and reach the targets despite the PNA length of 70 mm, as observed by clinical and electrophysiological evaluations of outcomes. This distance was even longer than previously reported (31). Whether tacrolimus is an option in PNA reconstruction in humans remains to be elucidated. Such a treatment should be balanced against the potential side effects of the pharmacological substance. Whether pain-related genes are down-regulated in dorsal root ganglia (DRG), as observed in experimental studies of acellular nerve allografts (35), are now known. Interestingly, two of the present individuals reported no analgesic use. However, a remaining (severely painful) Tinel sign was reported particularly in one of the individuals with a reconstructed neuroma in the median nerve. This is not surprising as a Tinel sign may even be observed after nerve reconstruction using conventional techniques, although the severity may vary.

In the two individuals with digital nerve injuries, the PNA was ≥25 mm, a length reported to be achievable for a PNA (i.e., average 35 mm; range 25–50 mm) in which axons can regenerate across and reach targets in skin (31, 44). In digital nerve neuroma, an alternative technique, mentioned as “relocation nerve grafting” (i.e., use of PNAs from an excised neuroma to reach interosseous muscles), has been described (45). However, this was not necessary in any of our individuals since we were able to attach the PNA to the distal nerve end. Touch perception, measured by SWM and interpreted as diminished protective sensation and diminished light touch, was found in the finger pulps after the present digital nerve reconstructions. Surprisingly, even detectable 2-PD, as well as recovered motor function in some median nerve innervated muscles with EMG signs of reinnervation at 36 months, was observed after one of the median nerve reconstructions despite the long PNA (70 mm) and PNA surgery a long time after the initial injury. No objective EMG signs of muscle reinnervation of the small intrinsic muscles for the first dorsal interosseous and adductor pollicis muscles were observed after PNA reconstruction of the deep ulnar nerve branch, where the surgery was intended only to recover some function of the first dorsal interosseous and adductor muscles. However, improved grip and manual dexterity, resulting in less pain-discomfort-fatigue, were noted in this individual. Reinnervation of the small-sized intrinsic muscles is particularly difficult after a long denervation and atrophy (46). Therefore, we performed an additional anterior interosseous nerve (AIN) transfer as a procedure intended to facilitate motor reinnervation, which remains a debatable procedure (47, 48).

An iatrogenic injury was the cause in two of the individuals, and the PNA alternative may be a good option in such cases, since they may be reluctant to undergo more nerve surgery through the harvest of a sural or other nerve to get a nerve graft (31). In such individuals, a PNA is an applicable alternative to nerve reconstruction surgery, even if the morbidity of nerve grafts may be limited (49), but should be balanced against what can be achieved and the previous experiences of the affected individuals (31, 50).

## 4 Strengths and limitations

We acknowledge that we report only a few individuals reconstructed with PNAs for different neuromas in the upper limb, with a heterogeneity in follow-up procedures depending on the affected nerve. Thus, larger numbers of individuals with pain related to neuroma need to be treated to make a firm conclusion, as well as further clearly define which individuals with pain problems, or discomfort, benefit from surgery using this indication and surgical approach. Our data agrees with a recent report of a larger number of treated individuals (*n* = 21) with pain due to neuroma in digital and major nerves of the upper (24) and the lower limbs (26). In addition, together with these articles, an additional recent report with 21 individuals of neuroma treated with PNA (31), and the present individuals, the overall data highlights the use of PNAs in neuroma surgery with potential improvement of the health situation in the affected individuals with painful neuroma. Other aspects, including socioeconomic position and comorbidities, which may impact outcomes and pain problems, were not focused on in the present study (51–53). Due to the heterogeneity of individuals with pain problems after neuroma, information about outcomes for any individual is valuable as it may serve as a basis for evaluating individuals in a systematic review, a meta-analysis or a review of many individuals. No control group, as in a previous report (24), was included in this case series; instead, each individual served as its own control with preoperative status related to the specific injured nerve. In addition, the study was conducted in a tax-funded health care sector without any connection or discussions with any insurance company or with the producer or supplier of the used PNA. This is an important aspect in view of potential differences in outcomes of nerve repair or reconstruction after different types of nerve injuries presented in industry-supported papers and non-industry-supported articles (23, 28, 44, 54, 55).

## 5 Conclusion

We conclude that reconstruction of painful neuromas with PNAs may be a suitable option even long time after the nerve injury in selected individuals where autologous nerve reconstruction is not considered for other reasons, as it can relieve pain and enhance function.

## Permission to reuse and copyright

Permission was asked and was granted from Elsevier to publish an image in Figure 1 from Video 22.3 in Farnebo S, Thorfinn J, and Dahlin LB; Peripheral nerve repair and reconstruction (Chapter 22); in Neligan and Chang Plastic Surgery. Hand and Upper Limb, Sixth Edition. Pages 526–551, 2024.

## Data Availability

The datasets presented in this article are not readily available because Public access to the data is denied by the Swedish Authorities (Public Access to Information and Secrecy Act; https://www.government.se/information-material/2009/09/public-access-to-information-and-secrecy-act). Data can be made available for researchers after a special review that includes approval of the research project by both the National Ethics Committee (http://www.etikprovningsmyndigheten.se/en) and the local authorities' data safety committees. Requests to access the datasets should be directed to etikprovningsmyndigheten.se.
